# Feedback Control of a Nonlinear Electrostatic Force Transducer

**DOI:** 10.3390/s20247337

**Published:** 2020-12-21

**Authors:** Ivan Ryger, Richard Balogh, Stefan Chamraz, Alexandra Artusio-Glimpse, Michelle Stephens, Paul A. Williams, John Lehman

**Affiliations:** 1 Department of Physics, University of Colorado, Boulder, CO 80309, USA; om1air@gmail.com; 2 National Institute of Standards and Technology, Boulder, CO 80305, USA; alexandra.artusio-glimpse@nist.gov (A.A.-G.); michelle.stephens@nist.gov (M.S.); paul.williams@nist.gov (P.A.W.); john.lehman@nist.gov (J.L.); 3 Faculty of Electrical Engineering and Information Technology, Slovak University of Technology in Bratislava, Ilkovicova 3, 812 19 Bratislava, Slovakia; stefan.chamraz@stuba.sk

**Keywords:** capacitive transducer, mechanical resonance, nonlinear control

## Abstract

We document a feedback controller design for a nonlinear electrostatic transducer that exhibits a strong unloaded resonance. Challenging features of this type of transducer include the presence of multiple fixed points (some of which are unstable), nonlinear force-to-deflection transfer, effective spring-constant softening due to electrostatic loading and associated resonance frequency shift. Furthermore, due to the utilization of lowpass filters in the electronic readout circuitry, a significant amount of transport delay is introduced in the feedback loop. To stabilize this electro-mechanical system, we employ an active disturbance-rejecting controller with nonlinear force mapping and delay synchronization. As demonstrated by numerical simulations, the combination of these three control techniques stabilizes the system over a wide range of electrode deflections. The proposed controller shows good setpoint tracking and disturbance rejection, and improved settling time, compared to the sensor alone.

## 1. Introduction

This paper describes the design of a closed feedback loop system of an electrostatic force transducer for multi-watt class precision optical power measurements. A detailed outline of the force sensor and its operation in the open loop may be found in [1].

One conclusion of that paper was the need for a closed feedback loop system to bypass the nonlinear deflection-to-capacitance transfer—an obstacle for many elements of such a device, traceable calibration being a critical example. Usually, designer avoid using a highly nonlinear capacitor in their electronics due to complicated calibration and reproduction of parameters.

In designing the controller for this nonlinear electrostatic force transducer, it was, therefore, our goal to minimize the intrinsic nonlinearities between input power and output signal. We did this by holding constant the plate spacing of the parallel plate capacitor electrodes with a corrective electrostatic force from the feedback controller.

**Figure 1:**
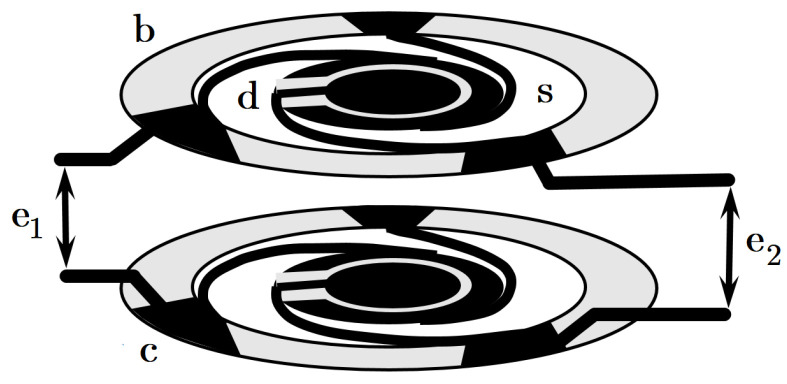
Schematic description of the electrostatic transducer subjected to control action. It consists of a moveable disk (d) with coaxial sensing and actuating electrodes, attached to the base (b) through spiral springs (s). Electrical connections are realized on contacts (c). The position sensing signal is fed to terminals (e${}_{1}$) and the electrostatic actuation is applied to terminals (e${}_{2}$). The geometrical separation of sensing and actuating electrodes minimizes the effect of direct capacitive crosstalk.

The device to be controlled (depicted in Figure 1) consists of a parallel plate capacitor with two moveable disks (d) attached to a rigid base (b) through spiral legs (s). By virtue of symmetric dual spring arrangement, any signal from common inertial forces (as vibrations and gravity) is greatly suppressed. The measured force is applied onto only one moveable surface, acting as a differential signal measured as the change of electrical capacitance. Though conceptually simple, controlling a single-ended capacitive transducer presents three important challenges in our design.

First, for the electronic bridge with a two plate-sensing capacitor, the output voltage to deflection transfer is highly nonlinear and governed mostly by the inversely proportional dependence of the transducer’s capacitance with respect to the capacitor plate spacing.

An interesting approach to this problem has been demonstrated by connecting the sensing capacitor as a variable impedance in the feedback branch of the switched capacitor transimpedance amplifier [2]. Unfortunately, this simple and cost-effective open loop approach cannot suppress the typical mechanical resonance of sensing capacitors with electrodes suspended on a spring. The electrostatic force between two surfaces is also non-linear with surface separation. To avoid these two problems, authors typically design their device with a three-plate differential capacitor [3,4,5,6,7,8,9] (eventually connected to a Bluemlein impedance bridge [5,10]). This scheme allows a reasonable degree of system linearization about a fixed point [11] under the assumptions that the magnitudes of perturbations are small and the control system acts instantaneously or with a small delay. In such a case, the excursions from the linearized regions are small enough, so the system parameters can be regarded to be constant and independent of the state of the system, allowing standard stability analysis methods (eigenvalue analysis of the system at critical points [11]) and linear controller architectures to be used ([3,4,12,13]). However, in our case, the area of the moveable electrode must be exposed to the force exerted by laser light. Thus, we are limited to a topology where a single-sided sensing capacitor is balanced against a reference arm capacitor, prohibiting us from benefiting from an already established linearization scheme. Furthermore, as we show later, due to the existence of a pull-in effect and strong deflection-dependent sensitivity to the variation in electrostatic force in conjunction with the high resonant quality of our capacitive sensor, these linearization methods are inapplicable for our problem.

Second, micromachined sensors exhibit high unloaded resonant quality factors, resulting in long exponentially-decaying oscillations and sharp phase steps in the transfer function frequency spectra. To compensate for this effect, researchers commonly introduce a notch filter that compensates for the step in the transfer function phase and equalizes the magnitude response. However, this approach can only be used under the assumption that the resonant frequency is constant. With electrostatic transducers, knowing the resonant frequency depends on an unknown initial spring position and applied electrostatic voltage due to the electrostatic spring softening effect [14,15]. Faced with varying system parameters, people refer to adaptive controllers that include a tracking notch filter or biquad filter [16] reacting to the changes of the system under control. However, if spring softening due to an electrostatic force is present, the reaction of the adaptive loop is slow compared to the almost instantaneous change of the resonant frequency, worsening the transient response and robustness of the system [16]. The state feedback controller architecture is an attractive alternative to the conventional approach (as in [4]). In such a controller, we may (in theory) arbitrarily place the poles/eigenvalues of the closed loop system to achieve a desired transient response, for example, suppressing resonance. In practice, the pole placement is constrained by the criteria of stability in the case of non-zero transport delay in the system. This approach also assumes all the internal states (namely, position, velocity and acceleration as a function of time) of the system under control are available for the state-feedback controller, which is not feasible for the type of capacitive transducer used in our sensor, because we only have access to the position measurement. Therefore, we employ a state observer (estimator) [16,17,18]. The state observer concept leverages knowledge of the applied control effort and the measured output from the system to infer the rest of the unavailable internal states. The external influence of the environment (even the forcing action that is our measurand) is treated as an unknown disturbance to compensate against. As described in [16], any unknown system characteristics are treated as disturbances by extending the state space of the state estimator [16,17]. In this treatment, accurate knowledge of the system’s physical parameters is not needed. Furthermore, parameters can change in time without a priori knowledge of the changing trend. The only tuning parameter of such a stabilizing controller is the control bandwidth. This active disturbance rejection controller (ADRC) concept has been confirmed to be reliable in many applications, including the dual-mass torque stabilization problem [16] and the amplitude stabilization of MEMS gyroscope vibrational modes [17].

Third, to achieve a sufficient signal-to-noise ratio, we must filter the transducer position signal. This frequency-selective component (analogue or digital filter), introduces group delay into the closed-loop system, causing instabilities in the feedback loop and impairing the transient response of the system. In common accelerometer applications, the filter transport delay is negligible since the system’s dynamics are usually slow relative to the time delay imposed on the system by filters ([3,4,5,8]). The problem of time delay in non-linear systems was also well described in [19,20]. If the loop transport delay is sufficiently small, it can be accepted by conventional feedback systems at the cost of affecting time-domain performance (overshoot, settling time), provided the stability criteria are met. If the time delay is considerable compared to the system’s dynamics, the loop may become unstable if the error signal arrives after a large enough delay such that the state of the controlled system has substantially changed. Then the controller effort may force the system into an unstable region instead of acting against a disturbance. Furthermore, in the case of state observers, the effect of delay is further amplified because it affects the state prediction. The most severe case is when a large time delay is introduced only in the system output’s measurement path. In such a case, the information about applied control action arrives at the state observer in time, whereas the system’s output measurement is delayed. Then the obtained state estimation progressively diverges from the real system states, leading to instabilities. To remedy this problem, a delay synchronization concept for the family of ADRCs was investigated in dissertations [21,22]. The control action information is intentionally delayed by the same amount as the measurement. Then, both signals arrive to the state observer synchronously, though delayed. Causality is not violated, and the observer calculates late-but-accurate information about the system states. The fact that this information is outdated does not need to be critical if the time delay meets the stability criteria for a closed-loop system. To address all the above-mentioned questions, we model an active disturbance rejection controller scheme with delay synchronization to stabilize the highly-nonlinear capacitive transducer in the presence of a transport delay. In Section 2, the equivalent model of the electrostatically-driven capacitive actuator is introduced. In Section 3.1, we analyze the stability regions of a capacitive sensor biased by a fixed electrostatic voltage. Upon this analysis, a stable working point for the sole sensor is suggested. Next, in Section 3.2, the perturbation analysis is applied to understand the natural resonant frequency shift of a capacitive transducer due to the application of electrostatic force. Then, in Section 4, the inverse of the voltage-force conversion is introduced to compensate the nonlinear height-dependent electrostatic force and consequently stabilize the feedback gain magnitude. As mentioned before, the noise-reducing lowpass filter is an integral part of the lock-in based position readout circuitry. For this reason, in Section 5 we investigate the closed loop small signal stability of the proposed feedback controller with delay introduced by this filter. Finally, in Section 6, the proposed controller structure is analyzed using the large signal transient simulation, proving its stability. Its performance is summarized in terms of speed, noise and linearity.

## 2. Nonlinear Capacitive Transducer Model

**Figure 2:**
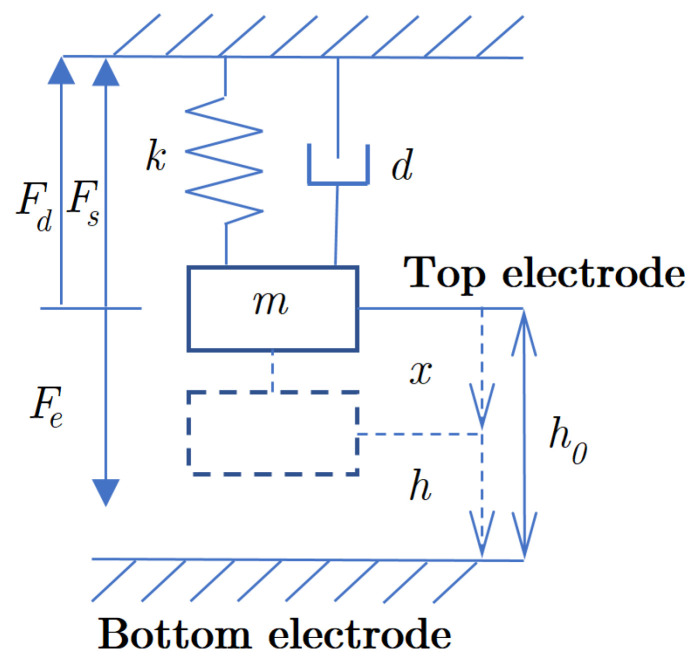
Equivalent mechanical diagram of our capacitive microactuator. The initial position of electrodes without electrostatic force is $h_{0}$; the deflected position (due to attractive electrostatic force) is *h*.

We represent our capacitive transducer (shown schematically in Figure 2) as a harmonic oscillator driven by the position-dependent electrostatic force ($F_{e}$). The relevant mechanical parameters of the system are the spring constant (*k*), the top electrode’s mass (*m*), and the damper (*d*) elements. In the steady state case, $F_{e}$ is compensated by the spring restoring force ($F_{s}$). We are using a dual spring differential arrangement [1], which allows us to neglect the influence of the common-mode gravitational force in the analysis. Common-mode forces pre-distort both springs equally; thus, the spacing between capacitor electrodes is kept independent of this force.

Using Newton’s 2nd law, we describe spring motion as

(1) $$F_{e}-F_{s}-F_{d}=ma,$$

where the acceleration *a* has the same direction as the electrostatic force $F_{e}$. Force $F_{d}=-d\dot{x}$ is the damping force. Based on the known vacuum permittivity $\varepsilon_{0}$ and the electrode area *A*, Equation (1) is rewritten into form

(2) $$-m\ddot{x}+d\dot{x}-kx+\left(1/2\right)\epsilon_{0}AV^{2}{\left(h_{0}-x\right)}^{-2}=0,$$

where symbols $h_{0}$ and *x* represent initial electrode spacing and deflection, respectively. The applied electrostatic voltage is denoted by variable *V*. The dot symbol in (2) denotes a time derivative.

## 3. Stability Modes of a Nonlinear Capacitive Transducer

Our electro-mechanical transducer has a highly nonlinear voltage to deflection conversion. For a given electrostatic voltage there are multiple fixed points at which an equilibrium between electrostatic attractive force and spring restoring force is established. However, some of these points are stable (the deflection converges toward them) and others are unstable (a perturbation causes the deflection to diverge). The following analysis will help us to determine the range of sensor deflections for which the system will be potentially controllable.

### 3.1. Static Analysis of Fixed Points

First we assume the static state, where the attractive force is compensated by a restoring spring force. From static force equilibrium, stability is described by

(3) $$-kx+\left(1/2\right)\epsilon_{0}AV^{2}{\left(h_{0}-x\right)}^{-2}=0.$$

By separating the variables *x* and *V* we get two functions that are equal at fixed points. The first is dependent on the electrostatic voltage *V*:

(4) $$y_{1}\left(V\right)=\epsilon_{0}A{\left(2kh_{0}^{3}\right)}^{-1}V^{2}.$$

The second dependent is related to deflection *x*:

(5) $$y_{2}\left(x/h_{0}\right)=\left(x/h_{0}\right){\left(x/h_{0}-1\right)}^{2}$$

**Figure 3:**
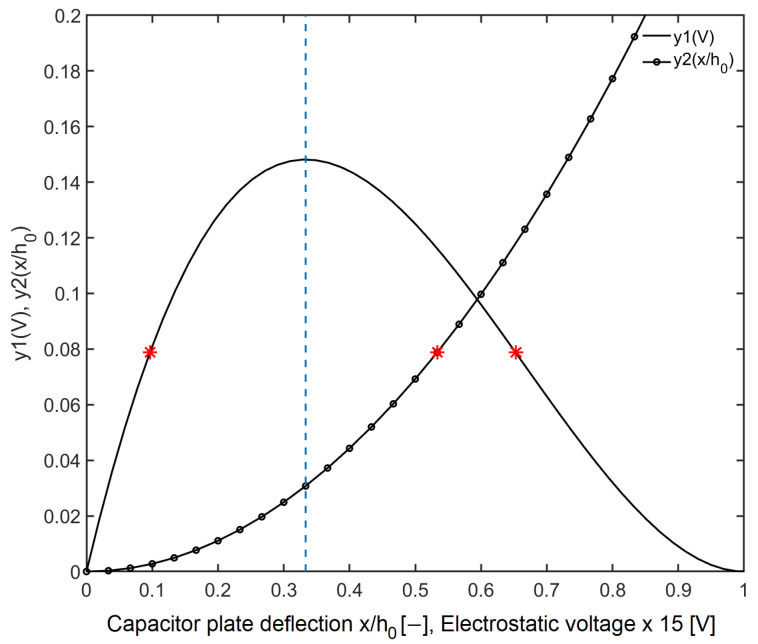
Plot of unitless functions $y_{1}(V$) and $y_{2}\left(x/h_{0}\right)$ upon their respective variables. The vertical dotted line divides the two stability regions. The asterisk (*) symbol denotes two height-bias points in the stable and unstable regions.

To get a pictorial view of solutions to Equation (3) we plot functions $y_{1}\left(V\right)$ and $y_{2}\left(x/h_{0}\right)$ into one graph (Figure 3). For plotting the quadratic function $y_{1}\left(V\right)$ we chose the parameters *k*, $h_{0}$ and *A* to be 65.8 N/m, 25.8 μm and 314 mm^2^, respectively. These values correspond to design parameters of our sensor. By relating $y_{1}=y_{2}$ for a given voltage *V*, we see that there are two possible solutions sitting on the curve $y_{2}$ (shown as red asterisk symbols). The function $y_{2}$ is a nonlinear polynomial, changing its derivative at the point $x=h_{0}/3$. This is a critical point that splits the range of deflections into two regions, of which the first is stable and the latter is unstable. These are separated in Figure 3 by a vertical dashed line.

To distinguish between stable and unstable bias points, a method demonstrated in [23,24] uses a small perturbation δx in deflection to determine if the restoring force acts against it. The total potential energy *E* of the spring pre-stretched by electrostatic force can be expressed as follows:

(6) $$E=-\left(1/2\right)\epsilon_{0}A{\left(h_{0}-x\right)}^{-1}V^{2}+\left(1/2\right)kx^{2}.$$

Then the restoring force *F* acting against a small perturbation is

(7) $$F=-\left(dE/dx\right)=\left(1/2\right)\epsilon_{0}A{\left(h_{0}-x\right)}^{-2}V^{2}-kx.$$

Differentiating (7) with respect to *x* and combining the result with (3), we get the expression for a virtual system stiffness:

(8) $$\kappa \left(x\right)=dF/dx=2kx{\left(h_{0}-x\right)}^{-1}-k$$

We can see that the stiffness changes its sign at the point $x_{p}=h_{0}/3$. For values below this deflection ($x<x_{p}$), the stiffness κ is negative, which implies the restoring force acts against the perturbation, thus stabilizing the position. Past the point $x_{p}$ the stiffness is positive, implying a divergent energy growth from any small perturbation causing pull-in instability.

### 3.2. Small Signal Dynamic Characterization

For electrostatically-driven capacitive sensors/actuators the sensitivity of the actuator deflection δx to small perturbations of the electrostatic voltage δV is affected both by the voltage setpoint value $V_{0}$ and height *h*. In the analysis we assume a small change in electrostatic voltage δV superimposed onto the static voltage $V_{0}$. The equilibrium in this context is to be understood as the height $h_{e}$ between sensor electrodes for a given static bias voltage $V_{0}$. The coordinate system can be then translated from the initial height $h_{0}$ to the equilibrium point $h_{e}$. We then use the deflection to match the new equilibrium point. The equilibrium point $h_{e}$, calculated from the balance between electrostatic force and spring restoring force (9), is used as a new coordinate center for the subsequent linearized model

(9) $$k\left(h_{0}-h_{e}\right)+\eta V^{2}=0,$$

where $\eta =\epsilon_{0}A/\left(2h_{e}^{2}\right)$. Consequently, the nonlinear dynamics in the absence of external forcing is approximated by the linear term of its Taylor series expansion:

(10) $$-m\delta \ddot{x}-d\delta \dot{x}-k\delta x+\eta V^{2}+2\eta V^{2}h_{e}^{-1}\delta x=0.$$

Assume a small perturbation δV superimposed on DC voltage $V_{0}$ applied to sensor electrodes. In that case, the voltage applied will be $V=V_{0}+\delta V$, where $\delta V\ll V_{0}$. The second assumption is $\delta h\ll h_{e}$. Then, by using the approximation ${\left(V_{0}+\delta V\right)}^{2}\approx V_{0}^{2}+2V_{0}\delta V$, the response of the system dynamics can be rewritten into form

(11) $$m\delta \ddot{x}+d\delta \dot{x}+\left(k-2\eta h_{e}^{-1}V^{2}\right)\delta x-\eta V^{2}=2\eta V_{0}\delta V,$$

where the right-hand side represents the small driving voltage perturbation and the left-hand side represents the dynamics of the system. We can note the component in brackets being in the units of a spring constant. The second term in the bracket is opposing the spring constant, effectively decreasing its value as the voltage $V_{0}$ rises. This is known as spring-constant softening in electrostatic systems [15].

By Laplace-transforming Equation (11), we get a transfer function between electrostatic perturbation and mechanical deflection in form (12). Here, we omitted the constant term, since it is time-invariant (it does not affect the frequency transfer), and in the case of the linearized model, the superposition theorem is valid such that

(12) $$\left[s^{2}+2\zeta_{e}\omega_{0e}\left(h_{0},V_{0}\right)s+\omega_{0e}^{2}\right]\delta x\left(s\right)=\eta m^{-1}V_{0}\delta V\left(s\right),$$

where $2\zeta_{e}\omega_{0e}=d/m$ and a bias-dependent angular resonant frequency is expressed as

(13) $$\omega_{0e}^{2}=k_{e}/m=\left(k-\epsilon_{0}AV_{0}^{2}h_{e}^{-3}\right)m^{-1}$$

**Figure 4:**
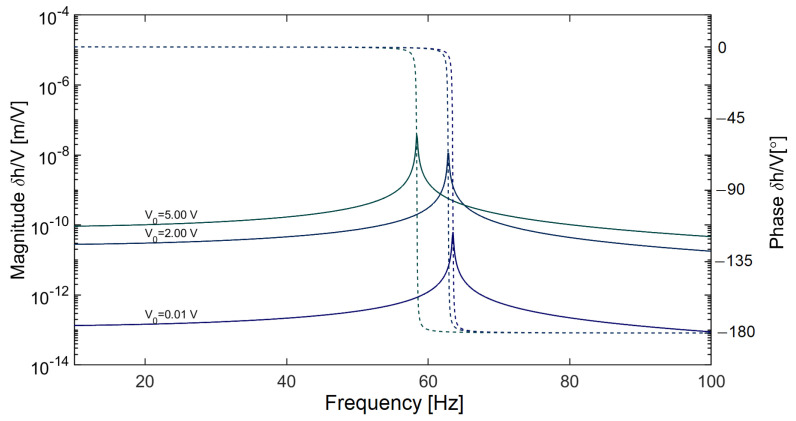
Frequency characteristics of the sensor’s mechanical response to a small perturbation in electrostatic bias, showing a considerable frequency shift of the resonance peak and a rise in sensitivity to the perturbation with rising electrostatic bias voltage. This shift is approximately quadratic. Solid curves indicate magnitude and dashed curves indicate phase.

The plot of small-signal sensitivity of the mechanical system to a perturbation in electrostatic bias voltage with a free parameter being the absolute value of electrostatic bias voltage is shown in Figure 4.

Here, we demonstrate the dependence of the mechanical resonant frequency on the applied electrostatic voltage. Note that the resonant frequency shifts toward lower values as the electrostatic force is increased. Furthermore, the transfer between perturbation in the electrostatic force and the mechanical response increases as the capacitor electrodes get closer (as a consequence of rising electrostatic force due to increased electrostatic bias voltage $V_{0}$). Hence, conventional linear feedback control methods (such as a proportional-integral-derivative controller with a fixed set of parameters) are likely to fail in this case, since the resonant frequency of the system and the transfer between the perturbation in electrostatic force and the deflection strongly depend on the state of the mechanical system and amount of applied electrostatic force. A sufficiently large external disturbance affects the electrode position *h* of the sensor, which in consequence drives the sensor out of the linearized region.

For this reason, we investigate the method of compensating the nonlinear electrostatic voltage to deflection transfer by back-mapping the applied electrostatic voltage *V* to the equivalent force *F* by known measured deflection *x*.

## 4. Compensation of the Voltage-Deflection Conversion Nonlinearity

The relationship between applied electrostatic voltage *V* and induced deflection *x* is first characterized by application of slowly varying ramp voltage onto moveable capacitor plates and simultaneous measurement of the electrode position interferometrically [1]. From this measurement we extract the transducer geometry-related constants *k*, $h_{0}$, *A* used in Equation (3). Consequently, an relationship between applied voltage *V* and height-dependent electrostatic force F(x,V) (2) is determined. Finally, the inverse of this relationship is applied in the internal structure of the feedback controller before output stimulus (electrostatic voltage) digital to analog conversion. Thereafter, the output signal from the state controller will be in physical units of force and the input action seen by the mechanical system will be again in units of force. Furthermore, the amplitude transfer between the electrostatic force perturbation and deflection will become independent of actual position. As shown later, this approach helps with stabilizing the system and allows us to use methods of linear system stability analysis. Using Equation (2) and replacing the true value of deflection *x* by its estimate from the state vector $\xi_{1}$, we can express the electrostatic voltage *V* in terms of the force *F* that is output from the state feedback controller

(14) $$V=\sqrt{2F/(\varepsilon_{0}A)}\;\left|h_{0}-\xi_{1}\right|.$$

Combining (14) with (2) yields an expression where the linear dynamic system is driven by a linear equivalent force. The problem that may arise in this case is if the estimate $\xi_{1}$ differs from true deflection *x*. This may occur shortly after system power on or if the rate of change in the input force disturbance is high due to the propagation delay and sampling time of the feedback controller. In such a case, for a brief moment the estimation error will be strongly dependent on this rate of change. For that reason, later in this article we will evaluate stability of the system by means of transient simulation, accounting for various sources of time delay, noise and nonlinearities.

## 5. Stability of the Linear Active Disturbance Rejection Controller with Transport Delay

**Figure 5:**
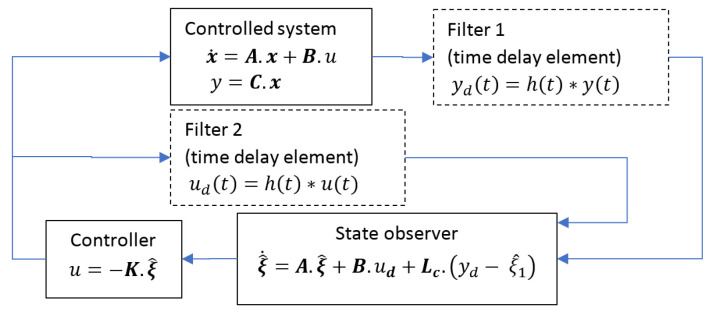
Block diagram of the sensor/state observer/state feedback controller scheme. This includes time-delay element 1 (low pass filter), which is an integral part of sensor preamplifier electronics. In order to provide a causal relationship between force and deflection information entering the state observer, a second time delay element identical to time delay element 1 is introduced. Here, the function h(t) denotes the impulse response of the filter and the asterisk denotes the convolution integral. This scheme is used to evaluate the stability of the feedback system.

As demonstrated in [16,17], the active disturbance rejection control appears to be an attractive solution for systems with uncertainty in dynamics (e.g., resonant frequency). It has been used recently for a similar problem with unknown time-variant dynamic properties of the system [16]. To a certain extent, it can handle nonlinear effects. We employed this particular state-observer/state feedback controller scheme in our approach and investigated its properties in conjunction with the aforementioned capacitive force sensor. The deflection signal from the controlled system (related to the electrical signal coming from bridge electronics) is lowpass filtered (Figure 5). This filter introduces a non-zero group delay into the signal path, affecting the system stability. As outlined in [21,22], to ensure causality between force and deflection signal, another identical filter will be introduced into force path (Filter 2 on Figure 5) of the state observer.

The transfer function analysis for a continuous time ADRC without time delay has been presented in [21,25]. The dead-time compensation was introduced into continuous-time models in [22], pp. 33–52. In this section, we derive the governing equations for the ADRC discrete time difference equations of a system affected by a non-zero loop delay introduced by discrete-time digital filters with arbitrary transfer function H(z). We analyze the stability of second order dynamic system driven by a linear force from the state feedback controller for a given shape of a lowpass transfer function H(z). In subsections A through C, the controller architecture is split into three main building blocks (state observer, feedback controller and sensor dynamics) with respective discrete time transfer functions finally combined into a closed loop system.

### 5.1. Extended State Observer

The extended state observer for the ADRC is defined as

(15) $$\dot{\hat{\xi }}\left(t\right)=A\hat{\xi }\left(t\right)+Bu\left(t\right)+L_{c}\left(y\left(t\right)-H\hat{\xi }\left(t\right)\right),$$

where the constant matrices A and B are dynamic and forcing matrices identical with the original ADRC design [17]

(16) $$A=\left[\begin{array}{ccc} 0 & 1 & 0\\ 0 & 0 & 1\\ 0 & 0 & 0 \end{array}\right]\;\mathrm{and}\;B={\left[\begin{array}{ccc} 0 & b^{-1} & 0 \end{array}\right]}^{T},$$

where $b^{-1}=m$ is again the mass. The constant vector $L_{c}$ represents the corrective gains ensuring that the estimated position will converge to its actual measurement. $L_{c}$ is designed using Ackermann’s formula [17] as

(17) $$L_{c}={\left[\begin{array}{ccc} 1-\beta^{3}, & \frac{3{\left(\beta -1\right)}^{2}\left(\beta +1\right)}{2T_{S}}, & -\frac{{\left(\beta -1\right)}^{3}}{T_{S}^{2}} \end{array}\right]}^{T}$$

where $\beta =\exp(\omega_{0}T_{s})$ is a constant dependent on observer bandwidth $\omega_{0}$. By transforming these equations into the discrete time domain, we get

(18) $$\hat{\xi }\left(k\right)=\bar{\xi }\left(k\right)+L_{c}\left[y\left(k\right)-H\bar{\xi }\left(k\right)\right].$$

Here the term $\bar{\xi }\left(k\right)$ represents the value of the system’s internal states calculated at the discrete time *k*. $T_{s}$ and the term in brackets represent the error-dependent corrective term that consists of measured scalar deflection y(k) and its estimate $H\bar{\xi }\left(k\right)$. The state vector estimation is calculated from the difference equation:

(19) $$\bar{\xi }\left(k\right)=\Phi \hat{\xi }\left(k-1\right)+\Gamma u\left(k-1\right),$$

where the constant matrices Φ and Γ represent the discrete time sampled transition matrix and forcing matrix [26] of the respective linear time model

(20) $$\Phi =e^{AT_{s}}$$

(21) $$\Gamma =\int_{0}^{T_{s}}e^{A\tau }Bd\tau =A^{-1}\left(e^{AT_{s}}-I\right)B$$

This yields

(22) $$\Phi =\left[\begin{array}{ccc} 1 & T_{s} & T_{s}^{2}/2\\ 0 & 1 & T_{s}\\ 0 & 0 & 1 \end{array}\right]$$

and

(23) $$\Gamma ={\left[\begin{array}{ccc} bT_{s}^{2}/2 & bT_{s} & 0 \end{array}\right]}^{T}$$

By combining (18) with (19) and *z*-transforming it into the frequency domain, we get

(24) $$\hat{\xi }\left(z\right)=\left(I-L_{c}H\right)\left[\Phi \hat{\xi }\left(z\right)+\Gamma u\left(z\right)\right]z^{-1}+L_{c}y\left(z\right).$$

Then, rearranging terms yields

(25) $$\left[I-(I-L_{c}H)\Phi z^{-1}\right]\hat{\xi }\left(z\right)=(I-L_{c}H)\Gamma u\left(z\right)z^{-1}+L_{c}y\left(z\right).$$

### 5.2. Feedback Controller

Due to non-zero transport delay in the feedback loop and finite sampling time of the observer the steady state-error will not be suppressed by the proportional-derivative regulator, as suggested in [16]. Thus, an additional integrator is needed to compensate that. The controller is then described by the following equation.

(26) $$u\left(z\right)=b^{-1}\left[K_{c}\left(rR-\hat{\xi }\right)-K_{i}\left(1+z^{-1}\right)\hat{\xi }\right]$$

The controller gain vectors $K_{c}$ and $K_{i}$ are chosen so that the continuous-time equivalent of the controller will satisfy the Hurwitz stability criterion [27]. For the ease of tuning, their coefficients are selected such that the characteristic equation will have a repeated root.

(27) $$K_{c}=\left[\begin{array}{ccc} 3\omega_{c}^{2} & 3\omega_{c} & 1 \end{array}\right],\;K_{i}=T_{s}/2\left[\begin{array}{ccc} \omega_{c}^{3} & 0 & 0 \end{array}\right].$$

### 5.3. Linear Dynamics

The linear part of the sensor’s dynamics without the nonlinear electrostatic forcing term in the state-space is

(28) $$\begin{array}{cc} \dot{x} & =A_{p}x+B_{p}u\\ y & =C_{p}x, \end{array}$$

where $x={\left[x_{1},x_{2}\right]}^{T}$ is the state vector that consists of position component $x_{1}$ and velocity component $x_{2}$. Variable *y* denotes the deflection as an output quantity being measured. Matrix $A_{p}$ defines the natural response of the system as

(29) $$A_{p}=\left[\begin{array}{cc} 0 & 1\\ -\omega_{0}^{2} & -2\zeta \omega_{0} \end{array}\right],$$

where $\omega_{0}^{2}=k/m$ is the natural resonant frequency of the sensor and $\zeta =d/2\sqrt{km}$ denotes the damping. The matrix $B_{p}$ defines the force coupling

(30) $$B_{p}={\left[\begin{array}{cc} 0 & m^{-1} \end{array}\right]}^{T}\;and\;C_{p}=\left[\begin{array}{cc} 1 & 0 \end{array}\right]$$

Again, the matrices $A_{p}$ and $B_{p}$ are brought into discrete time domain by transformations (20) and (21) yielding

(31) $$\begin{array}{cc} zx(z) & =\Phi_{p}x\left(z\right)+\Gamma_{p}u\left(z\right)\\ y(z) & =C_{p}x\left(z\right). \end{array}$$

Then the transfer between the input applied force and the output deflection is

(32) $$G_{p}=y\left(z\right)/u\left(z\right)=C{\left(zI-\Phi_{p}\right)}^{-1}\Gamma_{p}.$$

To evaluate the system (observer-state controller) transfer function between measured input deflection y(z) and output force u(z), we combine Equations (25) and (26) as follows.

(33) $$G_{c}=u\left(z\right)/y\left(z\right)={\left[1-K_{s}K_{o}K_{f}\right]}^{-1}K_{s}K_{o}L_{C},$$

where $K_{s}$ is the gain of the state feedback controller:

(34) $$K_{s}=-b^{-1}\left[K_{C}+K_{I}\left(1+z^{-1}\right)\right]$$

and matrices $K_{o}$ and $K_{f}$ define the state observer dynamics as:

(35) $$K_{o}={\left[I-(I-L_{C}H)\Phi z^{-1}\right]}^{-1}$$

and

(36) $$K_{f}=I-(I-L_{C}H)\Gamma z^{-1}.$$

In the case of finite impulse response (FIR) filters introduced to the feedback loop, their frequency dependent transfer is introduced as H(z). In that case, Equation (33) is modified into form

(37) $$G_{c}=u\left(z\right)/y\left(z\right)={\left[1-K_{s}K_{o}K_{f}H\left(z\right)\right]}^{-1}K_{s}K_{o}L_{C}H\left(z\right).$$

**Figure 6:**
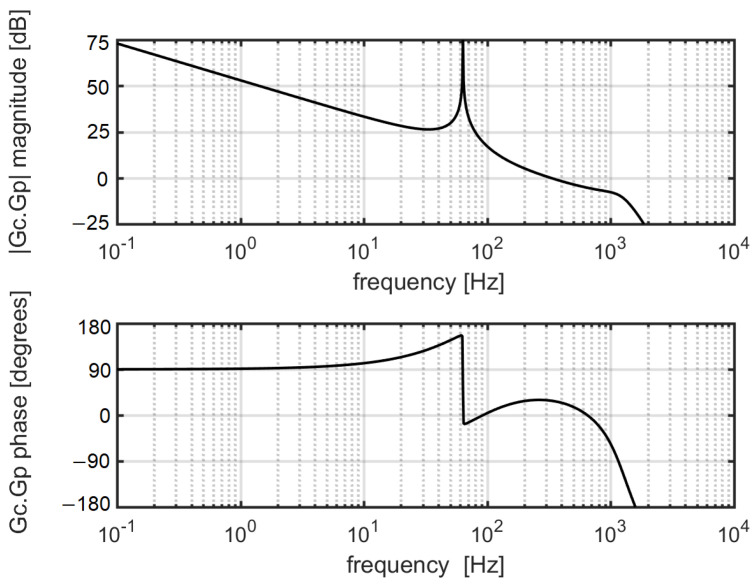
Stability simulation of an electrostatically-driven harmonic oscillator (with transfer function $G_{p}$ (32)) controlled by a discrete active disturbance rejection controller (ADRC; transfer function $G_{c}$ (37)). In the graph, the magnitude and phase of product $G_{c}.G_{p}$ are plotted. The sampling frequency was chosen to be $f_{s}=80\;\mathrm{kHz}$. The controller and observer bandwidth were chosen to be 200π and 2000π rad/s, respectively. A sinc-window FIR filter with 50 delay registers and a cutoff frequency of 1 kHz was introduced in both deflection and force path to the state observer.

Since the analytic solution defining the stability of this loop transfer function would be impractically complicated, we chose the graphic Bode plot evaluation. It shows that the open loop gain of the ensemble electro-mechanical sensor-observer-state controller has a 1/f roll off typical for an integrator and the phase stability margin at resonant frequency is about 23.8 degrees (see Figure 6).

## 6. Transient System Response Simulation

Since the exact nonlinear analysis of the system with multiple sources of nonlinearities would become exceedingly complex, the proposed controller concept was modeled in a simulation software. This approach helps us include the real circuit physical non-idealities in the model (quantization, signal saturation, noise) efficiently. For this purpose, we used the open source software Scilab/Xcos.

**Figure 7:**
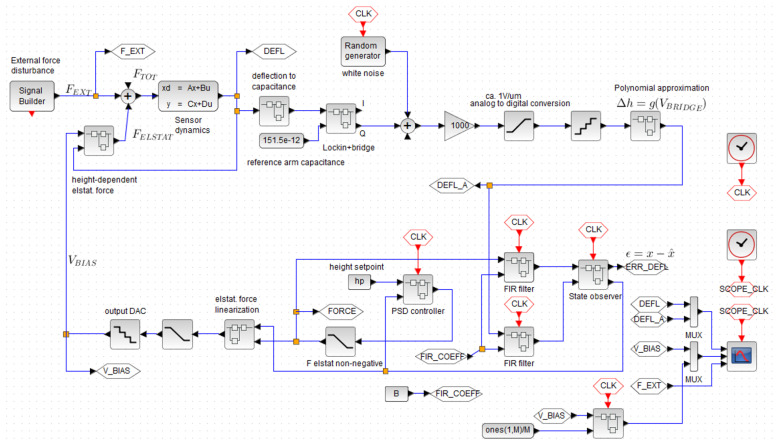
Block diagram of the sensor in a closed loop with the ADRC state feedback controller. The nonlinear electro-mechanical conversion’s effects on both deflection-dependent electrostatic force and capacitance are modeled. The function of synchronous lock-in demodulator is modeled by its behavioral model transforming the capacitance to the DC voltage through the magnitude and angle of the capacitive bridge AC transfer function. At the side of the digital controller, the sources of nonlinearities are modeled by saturation and quantization blocks (see the Supplementary Materials).

We approximate the capacitive bridge electrical output voltage by a polynomial to get the conversion between voltage and equivalent deflection. Then, the signal is FIR-filtered to reduce the magnitude of superimposed noise. Similarly, the estimated force signal is fed through an identical filter (to ensure causality between these two quantities [21,22]) and fed to an extended state observer [17]. The estimated state vector is fed to the feedback controller, where the deflection is compared with the height setpoint and the respective correction force is calculated. The electrostatic force is then back-mapped to the equivalent voltage in the block described by Equation (17). Finally, the calculated voltage is D/A-converted and fed as a corrective action signal to the actuator. The block diagram of the electro-mechanical sensor connecting with the digital controller is depicted in Figure 7.

**Figure 8:**
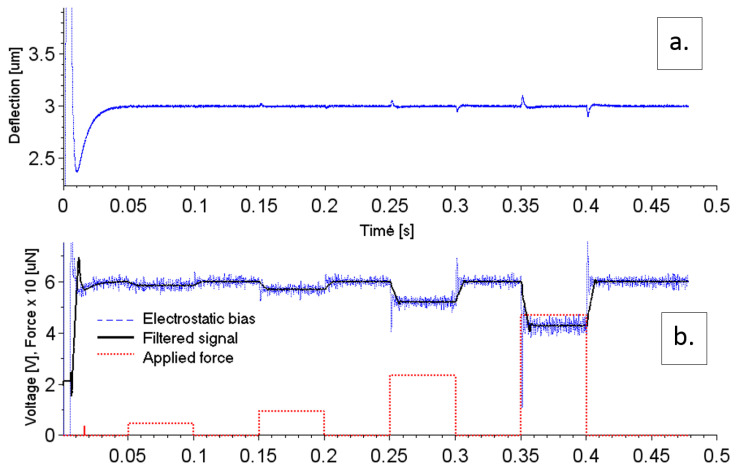
Transient response of the simulated closed feedback system to initial conditions (at the controller start-up) and application of a stepwise increase of external measured force. The initial ramp-up shows a strong overshoot in deflection signal caused by corrective controller action to a large initial difference between actual deflection and setpoint. The best achieved rise time is shown here. At lock, the sensor’s deflection (**a**) is kept constant by the compensating controller’s effort except for a short transient overshoot after application of the step force. The controller’s corrective effort (**b**) proportionally decreases as the input force increases.

According to the previous stability analysis, we selected the observer and controller loop bandwidths to be 2π×1000 and 2π×100 rad/s, respectively. The results of the time-domain simulation are displayed in Figure 8. At the beginning of the simulations, the deflection of the sensing capacitor’s plates first overshoots, and then at about 50 ms the deflection approaches the setpoint height, set to be $h_{p}=3\;\mu m$ (Figure 8a). This overshoot at the beginning of the simulation was caused by initial estimation of the deflection via state observer being different from the real sensor’s deflection (the time-delay registers in the deflection-path filter were initialized to zero and the capacitor plates were out of working range of the A/D converter and bridge amplifier). As long as the feedback controller adjusted the proper electrostatic capacitor plates’ distance, the overshoot caused by the disturbance was minimized. During simulations, we found the latter to be caused by the actuator non-linearity combined with the overall signal delay in the controller loop. After introducing a stepwise force disturbance, the capacitor plates went momentarily out of the operating point, followed by the delayed controller action. As a consequence, a slight signal overshoot was observed. The output signal rise time is defined by the overall feedback controller bandwidth. This problem is further discussed, e.g., in [16,17,18].

After the initial transient effect, the error vanishes, and the sensor’s capacitor plates are kept pre-deflected at a setpoint height by a certain amount of electrostatic voltage (Figure 8b). When a stepwise force with risetime $\tau_{r}=$ 10 μs is applied, the measured deflection first deviates from the setpoint. Then we see the controller reducing the amount of electrostatic voltage, compensating for the deflection error signal. This error then vanishes within about 10 ms time after application of the force step.

We note there is a strong noise component superimposed on the steady-state output electrostatic voltage. This is due to the relatively large controller bandwidth (necessary for the suppression of the mechanical resonance), since the force signal reacts with fast instantaneous fluctuations in the measured deflection signal. To decrease the noise magnitude, we post-filtered the measured electrostatic voltage with a 500-element moving average filter (Figure 8b).

The main source of the electrical noise is the capacitive bridge preamplifier itself. To limit it, we employed the post-filter as an integral part of the lock-in amplifier, processing the capacitive bridge signal. The second round of filtering was done by the feedback regulator itself—the higher the controller bandwidth, the better the stability (due to improved phase margin) and sensor mechanical resonance suppression, but it also causes higher susceptibility to noise. For that reason, the signal bandwidth within the control loop was set as a compromise. We found adding a post-filter to be a practical solution to improve the signal to noise ratio further, as the sampling rate of the ADRC must be kept high for good error tracking, but the required output signal data rate for this type of the mechanical sensor was an order of magnitude slower.

We took ten random samples of the steady-state output voltage for various input force levels to determine the mean and standard deviation of the voltage reading. The expected voltage at fixed deflection should obey equation

(38) $$\Delta F_{ES}=-F_{PH}=\frac{\varepsilon_{0}A}{2h^{2}}\left(V_{ES(0)}^{2}-V_{ES(F)}^{2}\right),$$

where $\Delta F_{ES},F_{PH},V_{ES(0)},V_{ES(F)},h$ represent the decrease in the electrostatic force, the measured force, electrostatic voltage before application of the force, electrostatic voltage bias with force present and sensing capacitor electrodes’ spacing, respectively. By calculating the difference of squares of voltage before and after application of the force step, we get a calibration factor that is proportional to previously defined geometrical factor η (see Table 1). The maximum nonlinearity of the calibration factor from this simulation was found to be around 0.79% and was at the level of system noise.

The measured data were post-filtered with a 500-tap boxcar FIR filter clocked at 80 kHz to improve the signal to noise ratio. This filter was also found to be the biggest contributor that determines the dynamics measurement, so there is a tradeoff between speed of the measurement and achieved detection limit. The risetime of the measurement signal from the simulation (Figure 8) was found to be about 10 ms. The noise floor in terms of equivalent power can be determined under assumption that the standard deviation of the voltage $\sigma_{V}$ (equivalent to RMS noise voltage magnitude) is much smaller than the electrostatic bias voltage $V_{ES(0)}$

(39) $$\sigma_{F}=\eta \left(V_{ES(0)}^{2}-{\left(V_{ES(0)}-\sigma_{V}\right)}^{2}\right)\approx 2\eta V_{ES(0)}\sigma_{V}$$

Evaluating this formula led us to an expected RMS noise floor of 0.028 μN, which corresponds to about 6 W RMS radiation pressure equivalent incident at 45° angle (see formula in [1]).

## 7. Conclusions

We demonstrated the design, simulation and analysis of a mechanical resonance suppressing controller for a single-side-driven capacitive microforce sensor. As we demonstrated in Section 3.1 for a given range of electrostatic voltages, this type of sensor has two fixed points, of which one is always unstable. Furthermore, if the bias deflection reduces to less than two-thirds of the initial plate spacing, the capacitor gets to the so-called pull-in region, where the electrostatic force diverges and the deflection is unstable. In Section 3.2 we showed that by increasing electrostatic bias voltage, the transfer between bias perturbation and deflection rises, and the natural resonant frequency of the spring-mass system shifts towards lower frequencies due to effect of the electrostatic spring softening. To partially compensate these two effects, we employed an electrostatic force to voltage back-mapping block into the feedback controller. We addressed the problem of unknown and time-variant system dynamics by investigating the application of active disturbance rejecting controller. Since the deflection measurement signal entering the state observer was delayed due to application of lowpass filters in the measurement path, we implemented a delay-synchronization concept to ensure the causal relationship between force and deflection signals. The stability of the feedback system was then analyzed in the discrete time domain using difference equations and the z-transform. By virtue of electrostatic force back-mapping, we could employ a Bode-plot stability analysis technique for finding the optimal controller and observer bandwidth. We analyzed the large signal behavior of the system by numerical simulation with multiple sources of nonlinearity, time delay, noise and saturation. The transient simulation was followed by numerical analysis of linearity, sensitivity and speed of the feedback system. As expected, the closed feedback loop helped in suppressing the resonance of the sensor, thereby improving the speed. The three critical features (ADRC, delay synchronization and force back-mapping) enabled controlling the entire feedback system in wide range of deflections. As a next step, we are planning to implement this scheme with a device such as that described in [1].

## Figures and Tables

**Table 1:** Calculation of the force calibration factor from the simulated force-dependent electrostatic voltage.

Force Step [μN]	0	4.7	9.4	24	47
Electrostatic voltage $V_{ES}$ [V]	6.012 ± 0.005	5.862 ± 0.007	5.710 ± 0.006	5.222 ± 0.005	4.296 ± 0.007
Calibration factor $\eta^{-1}$ [$V^{2}/\mu N$]		0.379 ± 0.030	0.377 ± 0.016	0.378 ± 0.005	0.376 ± 0.002

## Supplementary Materials

The code for the simulation methods introduced in this paper is available at IEEE Dataport http://dx.doi.org/10.21227/byg2-dq11.

## Disclaimer

This publication is a contribution of the U.S. government and is not subject to copyright. Identification of commercial items is for clarity and does not represent an endorsement by NIST.
